# Audit of Compliance With Stopping the Overprescribing of People With Autism Spectrum Condition (ASC) and Intellectual Disability (STOMP) Within the Child and Adolescent Mental Health Services (CAMHS) in Warrington, Mersey Care NHS

**DOI:** 10.1192/bjo.2024.597

**Published:** 2024-08-01

**Authors:** Vullong Kwarkas, Mariam Hanna

**Affiliations:** 1 Mersey Care NHS Foundation, Liverpool, United Kingdom; 2 Mersey Care NHS Foundation, Warrington, United Kingdom

## Abstract

**Aims:**

To assess compliance with the standards set in the Royal College of Psychiatrists (2021) Position Statement PS05/21: Stopping the overprescribing of people with ASC and Intellectual disability (STOMP) within the Child and Adolescent Mental Health Services (CAMHS) in Warrington, and Mersey Care Consent to Examination and Treatment Policy SD06

**Methods:**

A retrospective analysis of the electronic record of children and young persons (CYPs) having a diagnosis of either ASC, attention-deficit/hyperactivity disorder (ADHD), or both, and taking psychotropic medication while actively receiving care at Alders Warrington CAMHS between 1st May 2023 and 31st May 2023, was performed. The audit sample included 18 CYPs meeting the criteria, and we conducted the audit against 14 Compliance standards.

**Results:**

18 CYPs were included in the audit. 10 (55%) had a comorbid diagnosis of anxiety disorder, depression, or both, while eight (45%) had OCD, OCD Traits or Tic disorder. Four CYPs (22%) had challenging behaviour, including self-injurious behaviour in one of them. Although 17 (95%) of the CYPs had a mental disorder, the clinical indication for the psychotropic medication, which was documented for all patients, was also for behavioural problems viz challenging behaviour, and self-injurious behaviour, for 3 (17%) CYPs. For one patient (6%), there was no behavioural support plan (BSP), before the commencement of psychotropic medication. Three patients were prescribed psychotropic medication for behavioural problems. Two of the three patients with challenging behaviour had already commenced psychotropic medication before referral to the locality. All eligible patients had an initial multi-disciplinary team (MDT) meeting before prescription and routine 3-monthly reviews for efficacy and side effects. In all the cases, a specialist prescriber prescribed medication, and mental capacity was assessed and documented. Where necessary, a decision was taken in the patient's best interest. The service met all other requirements for compliance with standards set in the RCPsych position statement except for three criteria.

**Conclusion:**

Overall compliance with STOMP guidelines at the Alders Warrington CAMHS was 98%, with Significant Assurance. Dissemination of good practices and an early re-audit is strongly recommended.

